# Two cases of alopecic and aseptic nodules of the scalp

**DOI:** 10.1002/ski2.196

**Published:** 2023-02-23

**Authors:** Nicole Ufkes, Lauren Madigan

**Affiliations:** ^1^ Department of Dermatology University of Utah Salt Lake City Utah USA

## Abstract

We present two cases of alopecic and aseptic nodules of the scalp (AANS) that were previously misdiagnosed. AANS is characterized by solitary or multiple dome‐shaped alopecic nodules and predominantly affects young men. Dermatologists should be aware of this under recognized entity.

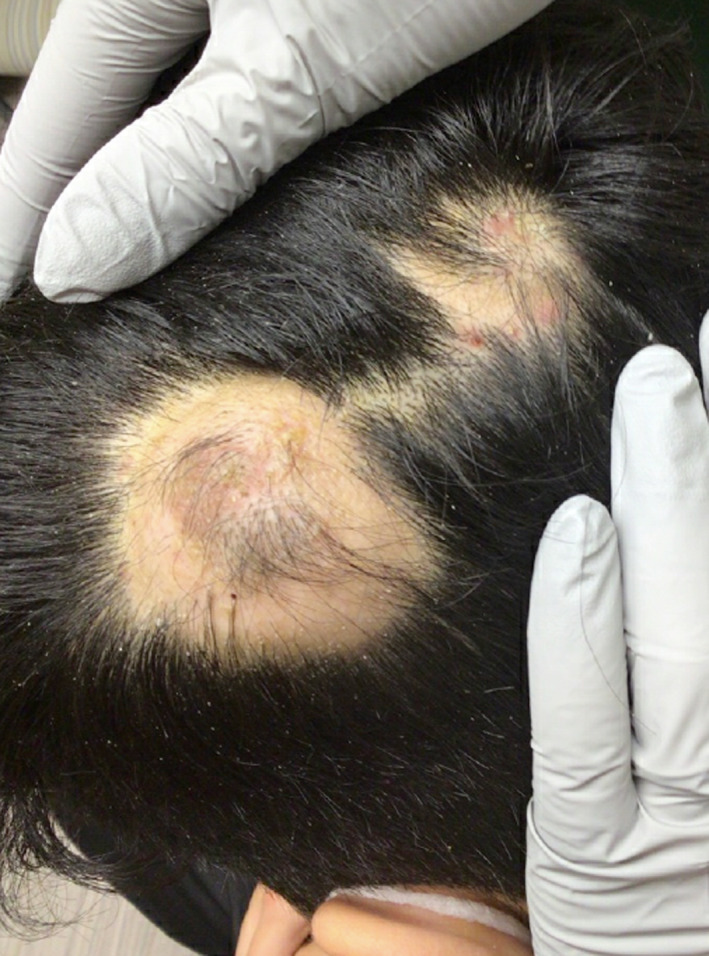

## 1 CASE PRESENTATION

**FIGURE 1:**
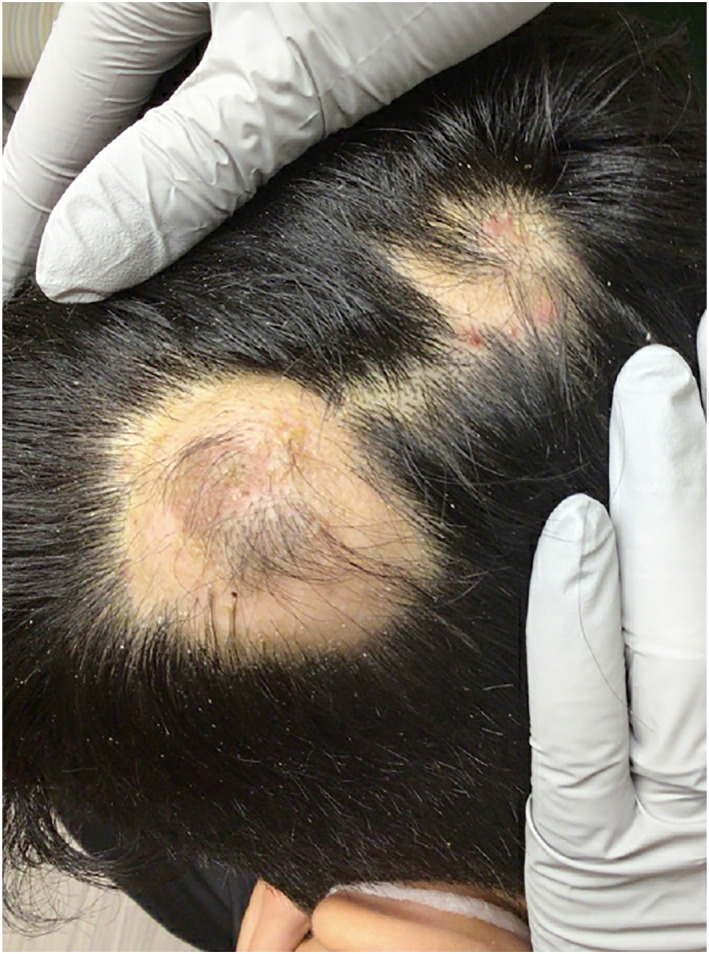
Two skin‐coloured nodules with overlying hair loss in patient one

**FIGURE 2:**
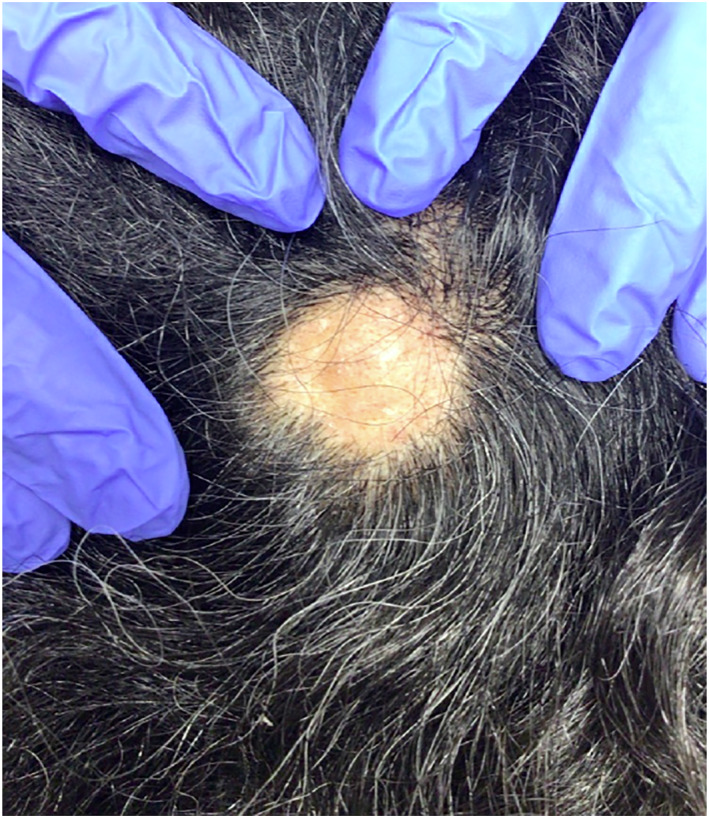
A boggy skin‐coloured nodule with overlying hair loss in patient two

**FIGURE 3:**
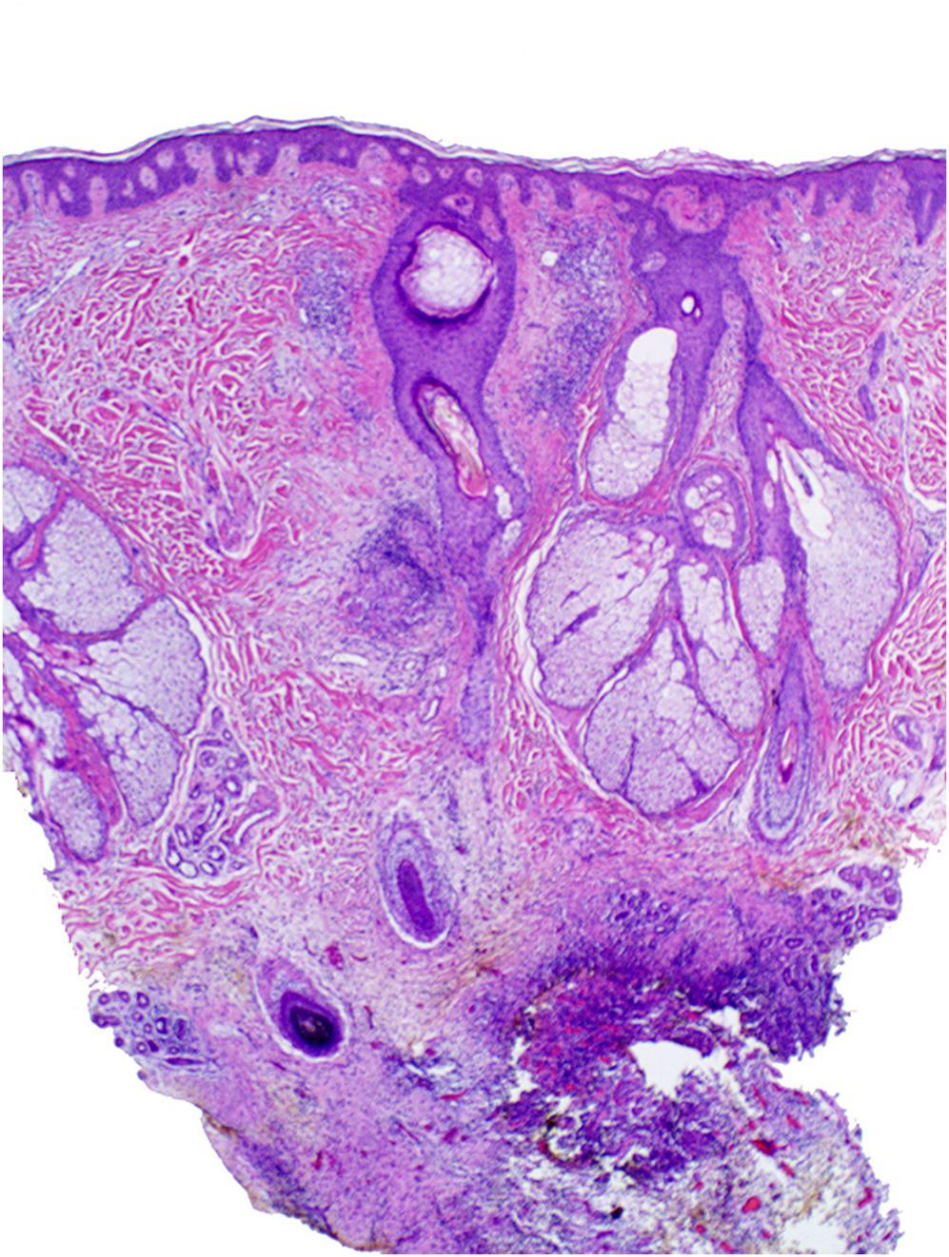
40x view showing perivascular and perifollicular infiltrates of lymphocytes, along with deep granulation tissue with dermal fibrosis and mixed infiltrates of lymphocytes, histiocytes and scattered multinucleated giant cells

**FIGURE 4:**
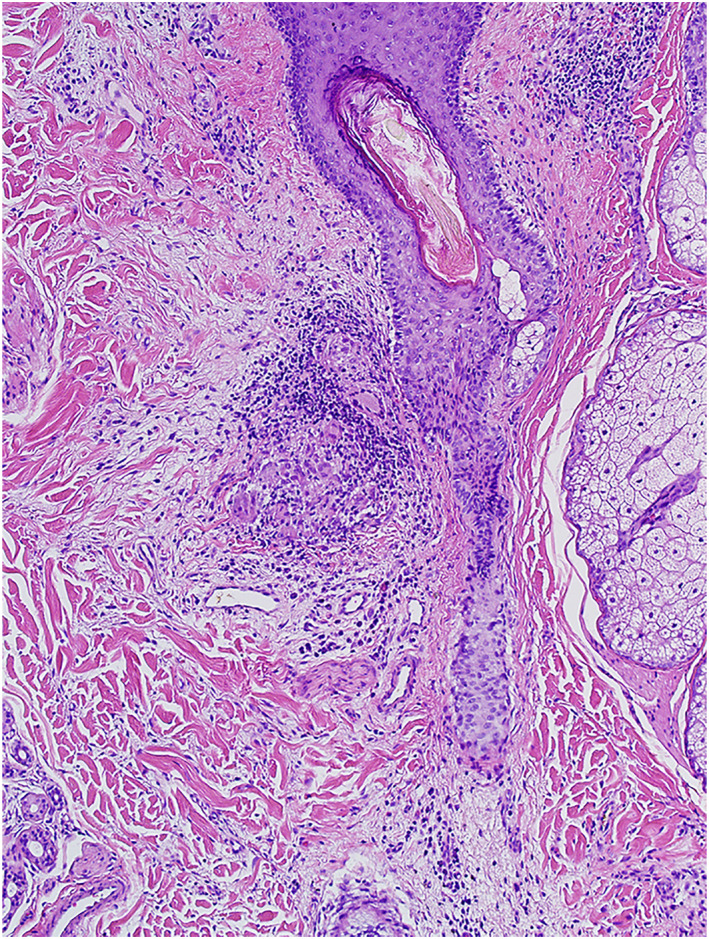
100x view showing a granulomatous infiltrate composed of scattered multinucleated giant cells and lymphocytes

**FIGURE 5:**
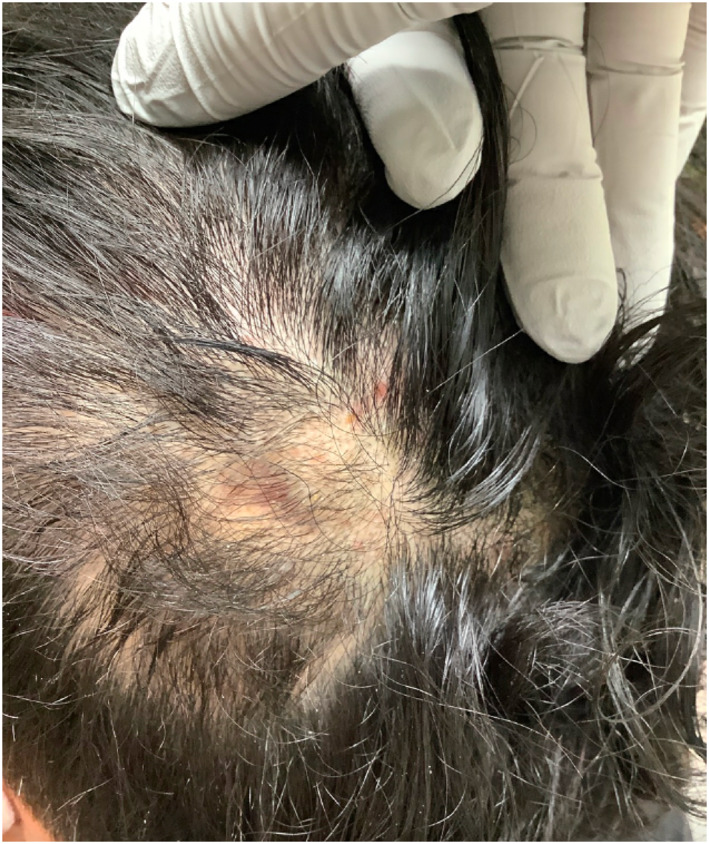
Resolution of the nodules with regrowth of overlying hair in patient one

Two healthy men ages 18 and 30 presented with painful scalp nodules and overlying hair loss. Both were previously treated for tinea capitis without improvement. Examination revealed focal areas of painful nodularity with associated fluctuance, crusting, and alopecia (Figures 1 and 2). Bacterial and fungal cultures were negative. One patient underwent punch biopsy which revealed deep fibrosis, granulation tissue with mixed inflammation, and granulomatous infiltrates composed of multinucleated giant cells and lymphocytes (Figures 3 and 4). A diagnosis of alopecic and aseptic nodules of the scalp (AANS) was made in both patients. AANS is an underrecognized inflammatory disorder characterized by single or multiple, skin‐coloured, dome‐shaped sterile nodules with overlying nonscarring alopecia. This entity has a predilection for young adult males with lesions typically located on the occiput and vertex. Trichoscopic findings include black and yellow dots, broken hairs, and fine vellus.^1^, ^2^ Treatment with doxycycline 100 mg/day for at least 3 months is efficient in most patients.^3^, ^4^, ^5^ Other options include repeated drainage and intralesional steroid injections. Some lesions will resolve spontaneously.^3^, ^4^, ^5^ Both patients described in this case notably improved after oral doxycycline and intralesional triamcinolone, with post‐treatment examination of one patient showing nodule resolution and overlying hair regrowth (Figure 5). The differential diagnosis of AANS often includes dissecting cellulitis of the scalp (DCS). However, in DCS, exam findings typically include pustules, papules, nodules, interconnecting tracts, and the development of cicatricial alopecia.

## CONFLICTS OF INTEREST

None to declare.

## AUTHOR CONTRIBUTIONS

**Nicole Ufkes**: Conceptualization (Equal); Writing – original draft (Lead); Writing – review & editing (Supporting). **Lauren Madigan**: Conceptualization (Equal); Supervision (Lead); Writing – original draft (Supporting); Writing – review & editing (Lead).

## ETHICS STATEMENT

Not applicable.

## Data Availability

Data sharing is not applicable to this article as no new data were created or analyzed in this study.
